# Functional Reconstruction with Latissimus Dorsi Flap following Resection of an Extensive Basal Cell Carcinoma in the Shoulder Region: A Case Report

**DOI:** 10.1055/s-0038-1660451

**Published:** 2018-06-11

**Authors:** Felix J. Paprottka, Dalius Klimas, Detlev Hebebrand

**Affiliations:** 1 Department of Plastic, Aesthetic, Reconstructive and Hand Surgery, AGAPLESION Diakonieklinikum Rotenburg, Rotenburg (Wümme), Germany; 2 Faculty of Medicine, Vilnius University, Vilnius, Lithuania

**Keywords:** basal cell carcinoma, shoulder defect, functional reconstruction, latissimus dorsi myocutaneous flap

## Abstract

Large and ulcerating skin tumors have become a rarity in the modern Western world. However, these conditions can cause serious life-threatening complications. The case of a 60-year-old male Caucasian patient is reported, who had suffered from an extensive basal cell carcinoma in the right shoulder region for several years. The patient kept the lesion secret from his friends and family and delayed presentation to health care services. After an episode of tumor-related heavy bleeding, the patient was referred to our clinic and received a radical surgical tumor resection—followed by defect coverage with a latissimus dorsi myocutaneous flap. An alternative treatment option that could be offered to the patient would have been a mutilating surgical procedure with an arm amputation. By using this plastic reconstructive surgical technique, the main function of the shoulder joint was conserved. The presented case demonstrates options for defect coverage of problematic wounds in anatomically complex body regions—like the shoulder—by using a functional reconstruction using myocutaneous flaps.

Advanced giant skin tumors with ulceration are rare in the modern medicine of the Western world. Therefore, these serious, disfiguring, and life-threatening conditions are typically found in developing countries. The aim of this publication is to demonstrate the different options for defect coverage of problematic wounds which are situated in anatomically complex regions and which demand good functional reconstruction. Hereby the authors present an exemplary case report.

## Patient Case

**Fig. 1:**
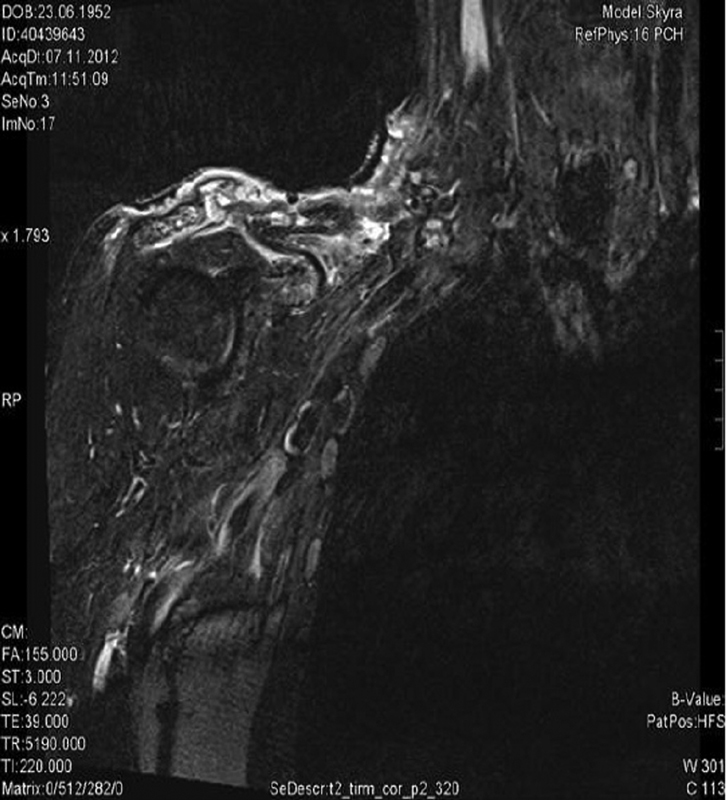
Preoperative magnetic resonance imaging (MRI) scan of the right shoulder for tumor staging. Large destructive basal cell carcinoma in the right shoulder region (TIRM sequence, T2, vertical axis). Complete destruction of the lateral end of the clavicle with surrounding soft tissue and trapezius, subclavian, and supraspinatus muscles.

**Fig. 2:**
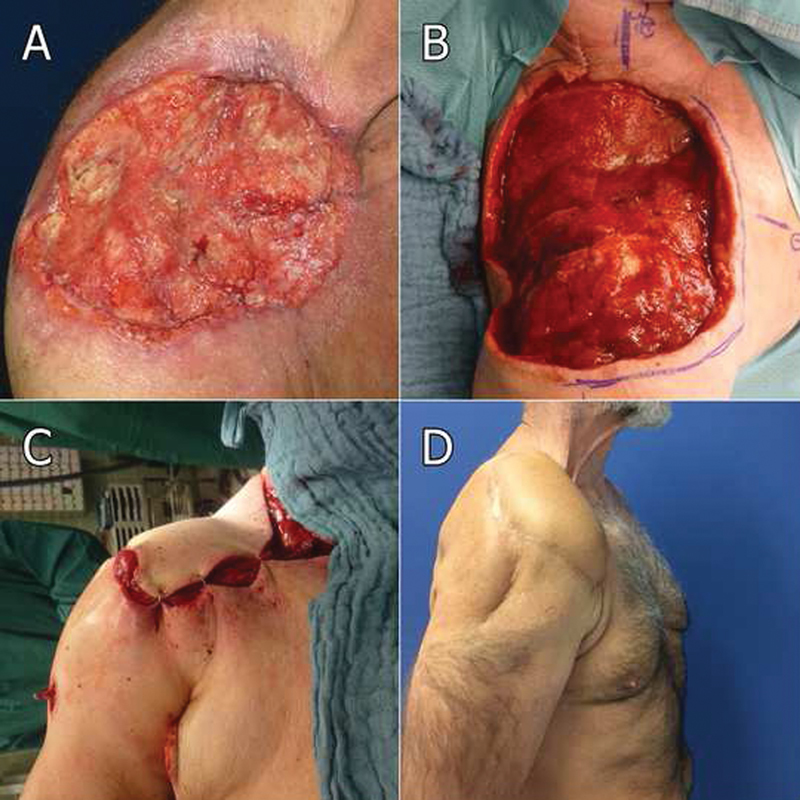
Ulcerating extensive basal cell carcinoma in the shoulder region before, during, and after surgery: ( **A** ) preoperative view—extensive basal cell carcinoma (15 × 15 cm); ( **B** ) intraoperative view with remaining defect after R0 resection status. Resection of the clavicle, acromion, right scapula, humeral fornix, and the humeral head with deltoid, trapezius, and supraspinatus muscles together with skin and subcutaneous tissue was performed; ( **C** ) intraoperative view with functional defect coverage using a latissimus dorsi myocutaneous flap (four-point fixation with nonresorbable suture material at the medial, lateral, dorsal, and ventral fascia of the remaining deltoid muscle); ( **D** ) one-year-follow-up after defect coverage with latissimus dorsi myocutaneous flap (LDMF).

A 60-year-old Caucasian male patient with an extensive basal cell carcinoma (BCC) (15 × 15 cm) in the right shoulder region was admitted to our clinic ( Fig. 1 ). According to his medical history, the patient had suffered from BCC, which had first occurred 20 years prior to his current presentation. At that time, it was treated with radiation with complete resolution of the tumor. Five years later, the patient unfortunately had recurrence of the tumor but refused further treatment. The patient kept the tumor recurrence secret from his relatives. From then on, the patient attempted to manage the constantly growing tumor conservatively himself. At the end of 2012, the patient suffered heavy bleeding from the tumor, and was at that point referred to our clinic. A tumor biopsy confirmed an ulcerating BCC. Magnetic resonance imaging scan showed complete destruction of the lateral end of the clavicle ( Fig. 2 ). No metastasis could be detected during further preoperative staging. Following multiple surgical procedures (totaling more than 10 operations), a subtotal resection of the clavicle, partial resection of the acromion, cranial, and dorsal parts of the right scapula, humeral fornix, and the humeral head with deltoid, trapezius, and supraspinatus muscles together with extensive skin and subcutaneous tissue resections were performed ( Fig. 1 ). After confirmed R0 resection status, the authors performed a functional defect coverage with a latissimus dorsi myocutaneous flap (LDMF) to restore movement of the shoulder joint ( Fig. 1 ). To do this, the authors harvested a right-sided pedicled LDMF with a large myocutanous island and did a four-point fixation for reliable functional reconstruction. Only 3 weeks after surgery the patient was already able to perform active flexion, extension, and 90° elevation of the right shoulder. The patient's mobility progressively improved with continuous daily physical therapy. Postoperative range of motion (1-year postoperative) is demonstrated in Table 1 .

**Table 1:** Shoulder movements (active and passive)

Range of motion	Neutral zero method (shoulder joint)	
	Right (plastic soft tissue reconstruction)	Left
Adduction/Abduction	20°-0–125° (passive: 30°-0–170°)	20°-0°–175° (passive: 20°-0–190°)
Anteversion/Retroversion	140°-0–30°	160°-0–40°
Horizontal extension/Flexion	135°-0–40°	140°-0–50°
Internal/External rotation (adduction)	80°-0–35°	95°-0–50°
Internal/External rotation in 90° (abduction)	60°-0–60°	70°-0–70°

Note: Demonstration of range of motion after reconstruction of a large shoulder defect with latissimus dorsi myocutaneous flap, using the neutral zero method.

## Discussion

BCC is the most common type of skin cancer and typically develops on sun-exposed areas. 1 While the BCC has a very low metastatic risk, the tumor can cause significant disfigurement by invading surrounding tissues. 2 BCC has many clinical subtypes—the most malignant one being the ulcus terebrans. It is characterized by ulceration, invasion, and destruction of the surrounding tissues. 2 Small basal cell tumors are regarded as relatively harmless, but the large and ulcerating carcinomas can become a surgical challenge with a less favorable prognosis. 2 In our presented case, the patient suffered from an advanced BCC (stage III) due to a protracted disease course.

The shoulder, together with the scapular complex, is anatomically defined as the junction between the trunk and the arm. 3 Wide tumor resection in the shoulder region often results in an extensive complex tissue defect, especially when progressing bone necrosis is involved. 3 Even if the limb is salvaged, wide excision are often associated with wound healing complications which can result in poor limb function. 3

To prevent possible complications and to maintain the best possible function of the shoulder joint, an advance planning of the surgical technique is essential. 4 As a highly mobile joint, the shoulder should be covered by stable and durable soft tissue to avoid motion limitations and loss of function. 5 To date, multiple authors have described reconstruction of isolated shoulder defects. In most cases, LDMF is used for defect coverage. Also, pedicled pectoralis major, trapezius, rectus abdominis, deltoid muscle flaps, and tensor fascia lata (TFL) free flaps offer interesting treatment options. 5 Ihara et al stated that the LDMF is the best option for reconstruction of large defects after extensive tumor resection within the shoulder region. 6 LDMF was first described for reconstruction after mastectomy, but today this flap is used in almost all sites of the body. 7 The advantages of the LDMF are its reliable large vascular pedicle with many cutaneous perforators, easy flap elevation, and minimal morbidity at the donor site. Furthermore, this flap does not require a microsurgical vascular anastomosis thereby resulting in higher success rates. 3 8 Alternative options used for shoulder reconstruction are less expendable, smaller in size, and are associated with greater donor site morbidity compared with the latissimus dorsi muscle (LDM). 6 Ihara et al believe that in certain situations the TFL flap could be the first choice for shoulder reconstruction—especially for deltoid replacement. 6 It is stated that the TFL muscle is more compact and is nearly comparable to the one of the deltoid muscle—whereas the LDM is too large. In our demonstrated case, TFL flap would not have provided an adequate defect coverage. Furthermore, the authors believe that the LDMF is a safer choice for such large defect zones.

Nevertheless, there are also some negative aspects concerning usage of the LDMF. Anatomically, LDM is a part of the shoulder girdle. Although, many publications state that the loss of LDM does not result in significant functional impairment, 9 10 Koh and Morrison revealed that LDM sacrifice may lead to more significant functional loss than previously documented. 9 A recent systematic review by Lee and Mun showed that limitations in shoulder joint after harvesting of LDMF could recover over time. 10 However, strength was reduced significantly and could not be recovered to the preoperative value even in the long run. 10 This needs to be taken into consideration in presurgical planning. In our presented patient case, the highest priority was the achievement of a negative margin (R0) resection of the tumor. Possible loss of postoperative range of motion was not taken into consideration while performing a radical excision. Recovery of range of motion is related to a successful LDMF transfer following intensive physiotherapy and biofeedback therapy.

## Summary

Plastic surgical reconstructive techniques do not only offer complete defect coverage of large defect zones, but also functional reconstruction with restoration of adequate range of motion. As demonstrated in our case report, this allows for successful reconstruction with a LDMF following wide tumor resection in anatomically complex regions such as the shoulder.
